# Treatment-Resistant Depression as a Heterogeneous Clinical Syndrome: A Personalized Medicine Framework for Diagnostic Reassessment, Suicide Risk, and Mechanism-Based Treatment

**DOI:** 10.3390/jpm16090468

**Published:** 2026-09-13

**Authors:** Davoud Amiri, Swetang J. Shah, Lamberto Briziarelli, Sara Amiri, Barry Karlsson

**Affiliations:** 1 Private Neuropsychiatric Practice, 753 10 Uppsala, Sweden; 2 Bay Area Community Health, San Jose, CA 95127, USA; swetang.dr@gmail.com; 3 Department of Public Health, University of Perugia, 06126 Perugia, Italy; lamberto.brizia@gmail.com; 4 Independent Researcher, 753 13 Uppsala, Sweden; saraamiri1997@gmail.com; 5 Department of Medical Sciences, Uppsala University, 751 85 Uppsala, Sweden; barry.karlsson@live.se

**Keywords:** treatment-resistant depression, personalized medicine, diagnostic reassessment, precision psychiatry, neuroplasticity, neuroinflammation, biomarkers, suicide risk, pseudo-resistance

## Abstract

**Background:** Treatment-resistant depression (TRD) is an operational category based on inadequate outcome after adequate antidepressant treatment. The same label may encompass insufficient treatment exposure, an incorrect primary diagnosis, clinically important comorbidity, or depressive illnesses with different maintaining mechanisms. This heterogeneity limits the clinical meaning of treatment history alone and complicates individualized care. **Objective:** To integrate evidence on diagnostic reassessment, suicide risk, neurobiology, psychological and functional factors, and treatment, and to define a clinically testable personalized medicine framework for TRD. Methods: Targeted PubMed/MEDLINE searches were conducted from database inception through 31 July 2026 and supplemented by reference-list searching. Two authors independently assessed publications for relevance, and disagreements were resolved by discussion and consensus. Ninety-six sources were included and synthesized narratively. **Results:** Inadequate exposure or adherence can produce pseudo-resistance, whereas bipolar spectrum illness, neurodevelopmental or trauma-related conditions, personality pathology, sleep or substance-related disorders, and medical disease may represent alternative diagnoses, comorbidities, or modifiers of treatment course. Glutamatergic signaling, impaired plasticity, stress-system dysregulation, inflammation, circadian disturbance, metabolism, and neural circuitry contribute to heterogeneity at the group level but do not define validated individual subtypes. Established pharmacological, psychological, and neuromodulatory interventions should be selected according to diagnosis, severity, urgency, previous response, comorbidity, functional impairment, safety, feasibility, and patient preference. **Conclusions:** TRD is an operational treatment-history category, not a unitary disease. A personalized medicine approach begins with confirmation of major depressive disorder, adequate treatment exposure and adherence, followed by parallel treatment of depression and clinically relevant comorbidity. Biomarkers should guide allocation only when they provide prospectively validated predictive value beyond high-quality clinical and functional assessment.

## 1. Introduction

Treatment-resistant depression (TRD) remains one of the greatest challenges in contemporary psychiatric practice. Despite substantial advances in pharmacological, psychological, and neuromodulatory treatments, a considerable proportion of patients with major depressive disorder (MDD) fail to achieve sustained remission after multiple evidence-based interventions. Persistent depressive symptoms are associated with marked functional impairment, diminished quality of life, increased healthcare utilization, substantial socioeconomic costs, and a markedly elevated risk of suicide. Consequently, TRD represents a major clinical and public health concern that continues to place a considerable burden on patients, families, and healthcare systems worldwide [1,2,3,4,5,6].

TRD is most commonly operationalized as failure to achieve remission after at least two antidepressant trials of adequate dose and duration, with adherence confirmed, during the same current depressive episode. Definitions nevertheless vary across research, regulatory, and clinical settings, particularly in how they specify treatment adequacy and outcome. Treatment history alone therefore does not establish etiology. Individuals meeting operational criteria may differ substantially in illness trajectory, psychiatric and medical comorbidity, functional impairment, biological features, and treatment responsiveness; TRD is better regarded as a heterogeneous clinical syndrome than as a single disease entity [1,2,3,4,5,6].

Treatment-refractory depression is not used as a synonym for TRD in this review. There is no universally accepted threshold for the term. As a pragmatic working descriptor, we reserve it for a more advanced stage within the TRD spectrum in which nonresponse persists after at least two adequate antidepressant trials, at least one evidence-based pharmacological augmentation strategy, and an adequate course of electroconvulsive therapy, unless electroconvulsive therapy is contraindicated, unavailable, or declined after an informed offer. The term is descriptive rather than a separate diagnosis, and every use should state the failed modalities and verified adequacy rather than rely on the label alone [1,2,3,4,5,6].

Persistent depressive symptoms do not necessarily indicate pharmacological resistance. Inadequate dose, duration, adherence, or drug exposure constitutes pseudo-resistance because an adequate treatment trial has not occurred. Previously unrecognized bipolar disorder may instead indicate an incorrect primary diagnosis. ADHD, autism spectrum disorder, post-traumatic stress disorder, personality disorder, sleep or substance-related disorders, and medical disease may coexist with correctly diagnosed major depression and modify its presentation, adherence, functional outcome, or treatment response. These categories should be distinguished rather than treated as equivalent explanations for failure [6,7,8,9,10,11,12,13,14,15,16,17,18,19,20,21,22,23,24,25,26,27,28,29,30,31,32,33,34,35,36,37,38,39].

Evidence reviewed here places the principal biological models of persistent depression within an integrated framework. Glutamatergic dysfunction, impaired synaptic plasticity, stress-system dysregulation, inflammation, disturbed sleep and circadian regulation, and metabolic abnormalities are supported in subsets of patients, but none defines TRD as a unitary biological disorder [40,41,42,43,44,45,46,47,48,49,50,51].

Suicidal ideation, attempts, and suicide mortality are more frequent in cohorts classified as TRD than in less treatment-resistant depression. This association should not be interpreted as proof that TRD itself causes suicidality: severity, illness duration, bipolarity, personality disorder, substance use, chronic pain, previous attempts, social adversity, and access to care may contribute to both treatment failure and risk. Repeated assessment must therefore address the full risk formulation rather than the TRD label alone [52,53,54,55,56,57,58,59,60,61,62].

The treatment literature is correspondingly heterogeneous. Evidence supports established augmentation strategies and selected rapid-acting or neuromodulatory interventions, whereas biomarker-guided treatment allocation remains investigational [63,64,65,66,67,68,69,70,71,72,73,74,75,76,77,78,79,80,81,82,83,84,85,86,87,88,89,90,91,92,93,94].

The aim of this structured narrative review is to synthesize current evidence regarding diagnostic reassessment, suicide risk, neurobiological mechanisms, psychological and functional modifiers, and mechanism-based treatment strategies in TRD. We propose a personalized medicine framework in which diagnostic clarification, verified treatment exposure, clinical phenotype, functional goals, safety, and patient preference guide current care, while biological measures are reserved for decisions in which they demonstrate added predictive value.

## 2. Methods

### 2.1. Review Design

This structured narrative review was designed to integrate evidence across five prespecified domains: diagnostic reassessment; neurobiological mechanisms; treatment selection; suicide risk; and biomarkers/precision psychiatry, with psychological and functional processes considered across the diagnostic and treatment domains. A narrative design was chosen because these domains address different clinical questions, populations, designs, and outcomes rather than one narrowly defined intervention comparison.

### 2.2. Literature Search

Targeted searches of PubMed/MEDLINE were conducted from database inception through 31 July 2026. For this revision, the searches were re-run on 10 September 2026 while retaining the prespecified publication-date cutoff. Six domain-specific Boolean strategies addressed definitions/diagnostic reassessment, differential diagnosis, mechanisms/biomarkers, treatment, suicide, and psychological/functional factors. The complete reproducible strings and the number of records returned by each search are reported in Supplementary Table S1. Reference lists of relevant systematic reviews, meta-analyses, guidelines, and pivotal trials were also examined for additional eligible publications.

### 2.3. Evidence Selection and Synthesis

Eligible sources were English-language human studies, systematic reviews, meta-analyses, evidence-based guidelines, randomized trials, and large observational or registry studies that directly informed a prespecified domain. Case reports, animal-only studies, opinion pieces without relevant evidence, duplicate or substantially overlapping reports without unique data, and publications without a clear connection to TRD or a designated differential/maintaining factor were excluded. Evidence from major depressive disorder populations not rigorously defined as TRD was retained only when it informed a mechanism or clinical question and is identified as indirect. Davoud Amiri and Swetang J Shah independently assessed titles/abstracts and, when required, full texts for relevance. Disagreements were resolved through discussion and consensus. Ninety-six distinct sources were included in the final synthesis. Because sources could inform more than one domain, the section-level counts overlap: 55 sources in diagnostic reassessment, 28 in neurobiology, 54 in treatment, 15 in suicide risk, and 17 in biomarkers/precision psychiatry.

### 2.4. Methodological Considerations

Data were synthesized narratively because the included questions, populations, interventions, comparators, and outcomes were not sufficiently uniform for a new pooled estimate. Where an included meta-analysis or trial reported a numerical effect, we extracted the effect measure, 95% confidence interval, and heterogeneity statistic when available, and interpreted clinical relevance alongside statistical significance and adverse-event burden. Selected quantitative estimates are presented in a dedicated summary table below. No de novo risk-of-bias assessment, certainty grading, meta-analysis, or PRISMA flow diagram was undertaken. The PubMed search counts in Supplementary Table S1 overlap and therefore must not be summed as a unique-record total; this is a structured narrative review rather than an exhaustive systematic review.

## 3. Diagnostic Reassessment

**Figure 1:**
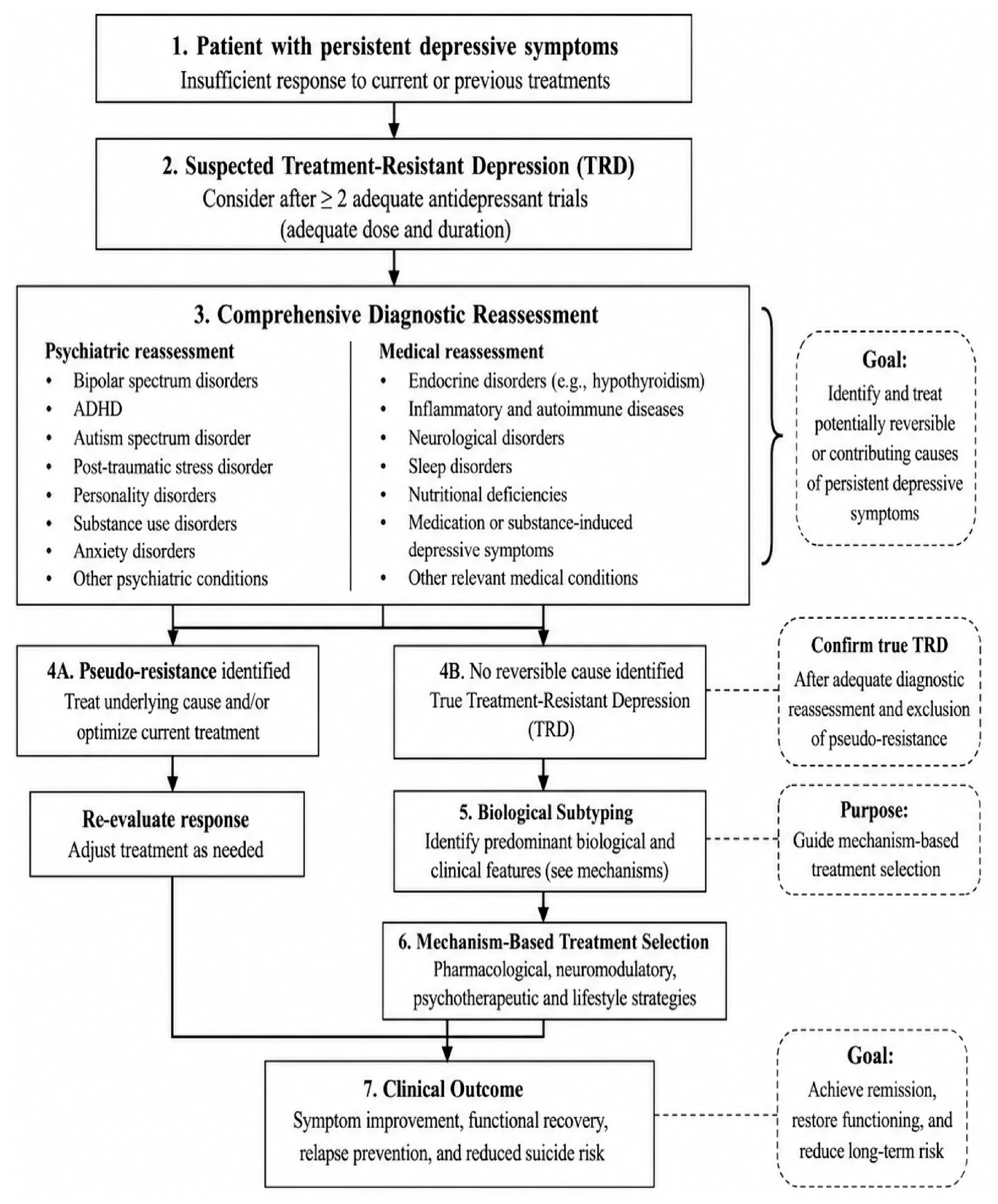
Clinical approach to suspected treatment-resistant depression. Treatment history alone does not establish true resistance. Adequacy, adherence, psychiatric and medical differentials, and reversible contributors should be reassessed before escalation. Phenotype and mechanism appraisal may then inform treatment priorities, but no biological subtype currently determines treatment selection in routine practice.

Diagnostic reassessment should follow a defined sequence rather than a broad search for possible causes. First, confirm that the current syndrome meets criteria for major depressive disorder and determine whether bipolarity, substance or medication effects, or a medical condition better explains it. Second, reconstruct each antidepressant trial within the same current episode, including dose, duration, adherence, exposure, tolerability, and measured outcome. Third, identify comorbidities and ongoing psychological, interpersonal, sleep, substance-related, and medical factors that modify the course. Only then should treatment resistance be classified and the next intervention selected. Table 1 summarizes the operational distinctions used in this review [1,2,3,4,5,6,12,15]. The overall clinical sequence is illustrated in Figure 1.

### 3.1. Bipolar Spectrum Disorders

Bipolar spectrum disorders represent one of the most important psychiatric differential diagnoses in patients with suspected treatment-resistant depression (TRD). A substantial proportion of individuals initially diagnosed with major depressive disorder are subsequently recognized as having bipolar disorder, particularly bipolar II disorder or other bipolar spectrum conditions in which hypomanic episodes are brief, subtle, or insufficiently recognized. Failure to identify bipolarity may result in repeated antidepressant trials with limited benefit, inappropriate classification of treatment resistance, delayed initiation of mood-stabilizing treatment, and an increased risk of antidepressant-induced mood destabilization. Accordingly, systematic reassessment for bipolar spectrum disorders is essential before a diagnosis of TRD is confirmed [6,7,15,18,19,20].

Several clinical characteristics should increase suspicion of underlying bipolar spectrum disorder in patients presenting with apparent TRD. These include early onset of depression, recurrent depressive episodes, postpartum depression, seasonal mood variation, psychotic depression, atypical depressive features, mixed symptoms, rapid mood fluctuations, antidepressant-induced hypomania, agitation or emotional instability, and a family history of bipolar disorder or suicide. Although none of these features is individually diagnostic, their cumulative presence substantially increases the likelihood that persistent depressive symptoms reflect bipolar illness rather than treatment-resistant unipolar depression [7,8,18,19,20].

Correct identification of bipolar spectrum disorder has major therapeutic implications. Whereas antidepressant monotherapy may contribute to mood destabilization, rapid cycling, or persistent depressive symptoms in susceptible individuals, recognition of bipolar depression allows implementation of evidence-based treatments including mood stabilizers, selected second-generation antipsychotics, and other guideline-supported interventions. Accurate diagnosis therefore influences treatment selection, prognosis, relapse prevention, and long-term clinical outcomes [7,18,19,20].

Bipolar spectrum disorder should be regarded as one of the most frequent and clinically important explanations for apparent treatment resistance. Comprehensive longitudinal assessment, systematic exploration of lifetime hypomanic symptoms, careful evaluation of family psychiatric history, and identification of mixed features should precede confirmation of TRD and escalation to more complex therapeutic interventions [6,15,18].

### 3.2. Adult Attention-Deficit/Hyperactivity Disorder (ADHD)

ADHD is relevant to reassessment because lifelong executive dysfunction, emotional dysregulation, and functional impairment can be mistaken for residual depression. It may also coexist with correctly diagnosed major depressive disorder, impair adherence, and alter the practical delivery of psychotherapy and pharmacotherapy. Assessment is warranted when attentional and organizational difficulties clearly predate the depressive episode, but the presence of ADHD does not invalidate a concurrent diagnosis of depression [9,21,22,23,24,25,91].

Several clinical characteristics should increase suspicion of previously unrecognized ADHD in adults presenting with apparent TRD. These include academic underachievement despite preserved intellectual ability, chronic procrastination, occupational instability, repeated failure to complete tasks, emotional dysregulation, impulsive decision-making, and longstanding interpersonal difficulties. Anxiety disorders, substance use disorders, and sleep disturbances frequently coexist and may further complicate the clinical presentation. Importantly, depressive episodes in individuals with ADHD often develop secondary to chronic functional impairment, repeated psychosocial stressors, and reduced self-esteem rather than representing isolated primary mood disorders [21,22,23,24,25,91].

Recognition of ADHD changes the formulation and may require psychoeducation, pharmacological treatment when indicated, behavioural strategies, environmental adaptations, and a more structured approach to adherence. Treatment of an active depressive episode should continue according to its severity and urgency; ADHD and depression are often managed in parallel, with sequencing determined by risk, impairment, and tolerability [9,23,24,25,91].

Adult ADHD should therefore be considered as a differential diagnosis, a comorbidity, or a modifier of treatment course, depending on symptom chronology. Developmental history and functional assessment determine which interpretation is justified [9,23,24,25,91].

### 3.3. Autism Spectrum Disorder

Autism spectrum disorder (ASD) may be recognized only after repeated treatment for depression, particularly in adults without intellectual disability. Longstanding social-communication differences, sensory burden, and need for predictability can be mistaken for residual depressive symptoms, but ASD commonly coexists with major depression. Its recognition should refine, rather than automatically replace, the depressive diagnosis [10,26,27,28,29,92].

Several clinical features should increase suspicion of underlying ASD in adults presenting with apparent TRD. These include lifelong social communication difficulties, preference for routines and predictability, restricted interests, sensory hypersensitivity, discomfort with change, chronic interpersonal difficulties, and a history of feeling different from peers since childhood. Anxiety disorders, obsessive compulsive symptoms, ADHD, and sleep disturbances frequently coexist and may further complicate the clinical presentation. Structured diagnostic instruments may support the assessment but should not replace a comprehensive clinical evaluation based on developmental history and current functional impairment [10,27,28,29,92].

Recognition of ASD has important implications for clinical management. Although depressive episodes should be treated according to established clinical guidelines, identification of the underlying neurodevelopmental condition allows more individualized interventions, including psychoeducation, environmental adaptations, structured support, and consideration of sensory and communication needs. Appropriate recognition of ASD may improve treatment engagement, functional outcomes, and overall quality of life while reducing the likelihood that persistent symptoms are incorrectly interpreted as treatment-resistant depression [15,28,92].

ASD should therefore be considered as a differential diagnosis, comorbidity, and treatment modifier. Developmental assessment clarifies the diagnosis, while sensory, communication, and cognitive needs guide adaptation of depression treatment [10,28,29,92].

### 3.4. Post-Traumatic Stress Disorder and Complex Post-Traumatic Stress Disorder

PTSD and CPTSD represent important differential diagnoses in patients presenting with persistent depressive symptoms and suspected TRD. Trauma-related disorders frequently coexist with depression and may remain unrecognized when clinical attention is focused primarily on mood symptoms. Consequently, patients with undetected PTSD or CPTSD may undergo multiple antidepressant trials with only partial improvement while the underlying trauma-related psychopathology remains untreated. Systematic assessment of trauma exposure and post-traumatic symptoms is therefore an essential component of the diagnostic evaluation before TRD is confirmed [11,35,36,37,38,39].

Several clinical features should increase suspicion of underlying PTSD or CPTSD in patients presenting with apparent TRD. These include recurrent nightmares, intrusive recollections, emotional numbing, exaggerated startle response, hypervigilance, avoidance behaviours, dissociative symptoms, chronic feelings of guilt or shame, and persistent interpersonal difficulties following traumatic experiences. A history of childhood adversity, interpersonal violence, military combat, torture, or repeated traumatic exposure should prompt further exploration of trauma-related psychopathology. Because many patients do not disclose traumatic experiences spontaneously, clinicians should inquire about trauma history in a sensitive and structured manner [11,35,36,37,38,39].

Failure to recognize PTSD or CPTSD has important therapeutic implications. Although antidepressants may reduce depressive symptoms in some individuals, persistent trauma-related psychopathology often requires trauma-focused psychological interventions as a central component of treatment. Accurate identification of PTSD or CPTSD therefore influences treatment planning, prognosis, and long-term functional recovery while reducing the likelihood that persistent symptoms are incorrectly interpreted as treatment-resistant depression [11,35,36,37,38].

PTSD and complex PTSD may be differential diagnoses or comorbid conditions. Trauma-focused treatment is often necessary, but an active major depressive episode should still receive appropriate treatment; urgency and suicide risk determine whether interventions are sequential or concurrent [11,35,36,37,38,39].

### 3.5. Personality Disorders

Personality disorders warrant careful consideration during the diagnostic reassessment of patients with persistent depressive symptoms and suspected TRD. Personality pathology frequently coexists with depressive disorders and may substantially influence symptom presentation, illness course, treatment adherence, interpersonal functioning, and clinical outcomes. Failure to recognize underlying personality disorders may contribute to persistent functional impairment, recurrent depressive episodes, and apparent treatment resistance despite otherwise appropriate management. Assessment of enduring personality traits and long-standing patterns of interpersonal functioning is an integral part of the comprehensive psychiatric evaluation before TRD is confirmed [30,31,32,33,34,93].

Several clinical characteristics should increase suspicion of clinically significant personality pathology in patients presenting with apparent TRD. These include longstanding interpersonal instability, chronic emotional dysregulation, recurrent self-injurious behaviour, unstable self-image, maladaptive coping strategies, persistent occupational or relational dysfunction, and repeated psychiatric presentations beginning early in adult life. Establishing whether these patterns preceded the onset of depressive episodes is particularly important because personality pathology represents an enduring vulnerability rather than an episodic mood disorder [30,31,32,33,34,93].

Recognition of coexisting personality disorders has important implications for treatment planning. Although depressive episodes should be managed according to established clinical guidelines, long-term improvement often requires psychotherapeutic interventions targeting emotional regulation, interpersonal functioning, and maladaptive personality traits. Identification of personality pathology may therefore improve treatment selection, strengthen the therapeutic alliance, and enhance long-term prognosis while reducing the likelihood that persistent symptoms are interpreted solely as evidence of treatment-resistant depression [15,30,31,34,93].

Personality disorders should not be treated as automatic explanations for antidepressant failure. They may coexist with major depression, shape interpersonal and emotional processes, affect engagement, and require longer-term psychological treatment. Longitudinal assessment should distinguish enduring patterns from state-dependent changes during a severe depressive episode [30,31,32,33,34,93].

### 3.6. Somatic Differential Diagnoses in Treatment-Resistant Depression

Medical assessment should distinguish common, treatable contributors from research-level mechanistic testing. History, examination, medication review, and the clinical phenotype determine which investigations are justified. A medical disorder may mimic depression, coexist with it, or worsen treatment response; these possibilities do not have the same diagnostic meaning and should be documented separately [1,6,12,15].

#### 3.6.1. Endocrine Disorders

Thyroid disorders can complicate diagnostic reassessment because their psychiatric and somatic manifestations may overlap with depression. Autoimmune thyroiditis and hypothyroidism are associated with depressive and anxiety symptoms, whereas hyperthyroidism may also present with anxiety, irritability, insomnia, cognitive dysfunction, or depression. Thyroid function testing is therefore a reasonable component of reassessment, particularly when symptoms, medical history, examination, or medication exposure raise suspicion [6,45,46,47].

Disorders of the HPA axis, including Cushing syndrome and, less commonly, adrenal insufficiency, have also been associated with depressive symptoms, cognitive impairment, and reduced quality of life. Chronic hypercortisolemia may contribute to hippocampal dysfunction, altered stress responsivity, metabolic disturbances, and impaired antidepressant response. Similarly, impaired glucose metabolism and diabetes mellitus have consistently been associated with an increased prevalence of depression, greater symptom severity, poorer functional outcomes, and reduced treatment responsiveness [48,49,50,51].

A complete blood count and thyroid-stimulating hormone are low-cost tests commonly considered during assessment; glucose or HbA1c, electrolytes, renal and hepatic function, and other endocrine tests should follow the history, examination, comorbidity, and planned treatment. Abnormal results require clinical interpretation and do not by themselves establish the cause of depression or treatment failure [6,15].

#### 3.6.2. Neurological Disorders

Neurological disorders constitute another important group of potentially reversible conditions in the evaluation of suspected TRD. Several neurological diseases may present with depressive symptoms as an early or prominent clinical manifestation, while others may contribute to persistent mood symptoms through cognitive impairment, chronic pain, fatigue, sleep disturbance, or neurodegenerative processes. Failure to recognize these conditions may result in misdiagnosis, delayed treatment, and apparent antidepressant non-response. Accordingly, a careful neurological history and examination should form part of the routine assessment, particularly when depressive symptoms are accompanied by cognitive decline, focal neurological signs, gait disturbance, seizures, or atypical clinical features [6,15,89,90].

Cerebrovascular disease has consistently been associated with late-life depression, and vascular depression has been proposed as a clinically distinct subtype characterized by executive dysfunction, psychomotor slowing, and reduced responsiveness to conventional antidepressant treatment. Likewise, neurodegenerative disorders, including Parkinson’s disease, Alzheimer’s disease, and other dementias, frequently present with depressive symptoms during the prodromal or early stages of illness. In these patients, depressive symptoms may reflect underlying neurodegeneration rather than primary affective illness, underscoring the importance of comprehensive neurological assessment before confirming TRD [89,90].

Neurological disorders should be regarded as important differential diagnoses rather than uncommon exceptions in patients with apparent TRD. Identification and treatment of an underlying neurological disease may substantially improve depressive symptoms and prevent unnecessary escalation to more complex psychiatric interventions [6,15].

#### 3.6.3. Inflammatory and Autoimmune Disorders

Inflammatory and autoimmune disorders may also contribute to persistent depressive symptoms and warrant consideration in selected patients with suspected TRD. A growing body of evidence demonstrates that chronic systemic inflammation and immune-mediated diseases may present with depressive symptoms before the underlying medical condition becomes clinically apparent or may substantially worsen the course of an existing depressive disorder. Consequently, failure to recognize these conditions may contribute to apparent antidepressant non-response and inappropriate escalation of psychiatric treatment. Careful review of systemic symptoms, inflammatory markers, medical history, and, when clinically indicated, targeted immunological investigations may therefore represent an important component of the assessment of patients with suspected TRD [6,15,43].

Several autoimmune diseases, including systemic lupus erythematosus, rheumatoid arthritis, Sjögren syndrome, inflammatory bowel disease, autoimmune thyroid disease, and celiac disease, have consistently been associated with increased rates of depressive disorders. Multiple mechanisms are likely involved, including chronic immune activation, increased production of pro-inflammatory cytokines, neuroendocrine dysregulation, chronic pain, fatigue, sleep disturbance, and psychosocial burden associated with chronic illness. In some patients, depressive symptoms may precede the diagnosis of the underlying autoimmune disorder, whereas in others they develop during periods of increased inflammatory disease activity. Recognition of these associations is therefore clinically important, particularly when depression occurs together with systemic symptoms such as arthralgia, recurrent fever, unexplained weight loss, gastrointestinal symptoms, skin manifestations, or persistent fatigue [43,73,74,75,76].

CRP or erythrocyte sedimentation rate should be requested when constitutional symptoms, examination findings, or known disease suggest inflammation. Autoimmune serology requires a specific clinical indication and is not a routine TRD screen. Cytokine panels, including IL-6 and TNF-α, remain research procedures: neither does a normal result exclude an inflammatory contribution nor does an elevated result confirm TRD or select a treatment [6,15,43,73,74,75,76].

#### 3.6.4. Nutritional and Metabolic Disorders

Nutritional and metabolic disorders represent another group of potentially reversible contributors to apparent treatment resistance. Deficiencies of essential vitamins and micronutrients, metabolic abnormalities, and chronic systemic disorders may contribute to persistent depressive symptoms, cognitive dysfunction, fatigue, and reduced responsiveness to antidepressant treatment. Because many of these conditions are readily identifiable through routine laboratory investigations and may improve following appropriate medical treatment, their recognition is an important component of the comprehensive assessment of patients with apparent treatment resistance. Accordingly, potentially relevant nutritional and metabolic abnormalities should be evaluated before TRD is confirmed or treatment is escalated [6,15].

Renal or hepatic disease and electrolyte disturbance may produce fatigue, cognitive impairment, sleep disturbance, and affective symptoms. Complete blood count, renal and hepatic function, electrolytes, and metabolic parameters are selected according to the clinical history, comorbidity, and treatment plan. Vitamin B12, folate, ferritin, and vitamin D should be tested when deficiency is plausible rather than as universal TRD biomarkers [6,15,48].

Preanalytical and biological variation must be considered when mechanistic measures are obtained. Sampling time, fasting status, acute infection, sleep, recent stress, body mass index, smoking, physical activity, medication, sample handling, and assay method can alter cortisol, cytokine, CRP, and metabolic results. Without standardized conditions and repeated validation, an observed difference may reflect measurement context rather than stable patient biology [51,73,74,75,76].

#### 3.6.5. Sleep Disorders

Sleep disorders are among the most common medical comorbidities in patients with depression and should be systematically evaluated during the diagnostic assessment of suspected treatment-resistant depression (TRD). Although insomnia is one of the diagnostic criteria for major depressive disorder, persistent sleep disturbance should not automatically be attributed to depression itself. Several primary sleep disorders may mimic depressive symptoms or contribute to persistent fatigue, cognitive impairment, reduced daytime functioning, and apparent antidepressant non-response. Consequently, failure to recognize underlying sleep disorders may lead to misclassification of pseudo-resistance and unnecessary escalation of psychiatric treatment. A careful sleep history is therefore an essential component of the clinical assessment before TRD is confirmed [6,15,41,42].

Obstructive sleep apnea (OSA) is one of the most clinically important and frequently underdiagnosed sleep disorders associated with depressive symptoms. Excessive daytime sleepiness, fatigue, impaired concentration, reduced motivation, and cognitive dysfunction may closely resemble persistent depression. Patients presenting with obesity, loud snoring, witnessed apneas, morning headaches, resistant hypertension, or excessive daytime somnolence should therefore be evaluated for possible sleep-disordered breathing. Appropriate diagnosis and treatment of OSA may substantially improve mood, cognitive functioning, and quality of life while reducing apparent treatment resistance [41,42].

Clinical evaluation should include assessment of sleep duration, sleep quality, excessive daytime sleepiness, snoring, witnessed apneas, restless legs symptoms, circadian preferences, shift work, medication use, and substance consumption. Sleep diaries, validated sleep questionnaires, actigraphy, or referral for polysomnography should be considered when clinical findings suggest an underlying sleep disorder. Routine polysomnography is not indicated for all patients with depression but should be performed when obstructive sleep apnea or other primary sleep disorders are clinically suspected [41,42].

#### 3.6.6. Medication- and Substance-Induced Depression

Medication- and substance-induced depressive symptoms represent an important and potentially reversible cause of apparent treatment-resistant depression (TRD). Numerous prescribed medications, alcohol, and recreational substances may precipitate depressive symptoms, exacerbate an existing depressive disorder, or reduce the effectiveness of antidepressant treatment. Failure to identify these contributing factors may result in inappropriate classification of pseudo-resistance and unnecessary escalation to more complex psychiatric interventions. A comprehensive review of prescribed medications, over-the-counter drugs, substance use, and treatment adherence is therefore required as part of the diagnostic reassessment of suspected TRD [6,15].

Several classes of medications have been associated with depressive symptoms, although the strength of evidence varies considerably. Corticosteroids are among the best-established pharmacological causes of mood disturbances and may induce depressive symptoms, emotional lability, anxiety, or cognitive impairment, particularly during prolonged treatment or at higher doses. Interferons and certain immunomodulatory agents have likewise been associated with depression in susceptible individuals. In contrast, evidence linking beta-blockers to depression remains inconsistent, and current data do not support a strong causal relationship for most patients. Nevertheless, medication-induced depression warrants particular attention when symptoms develop shortly after initiation or dose escalation of a potentially implicated drug [6,15,44].

Alcohol misuse remains one of the most common reversible contributors to persistent depressive symptoms and poor antidepressant response. Chronic alcohol consumption may worsen mood, disrupt sleep, impair cognitive functioning, and reduce treatment adherence. Similarly, cannabis, opioids, sedative-hypnotics, and other psychoactive substances may contribute to depressive symptoms through direct neurobiological effects, withdrawal phenomena, or secondary psychosocial consequences. Substance use disorders require active assessment, particularly in patients with fluctuating symptoms, recurrent relapse, or unexpectedly poor treatment response [6,15].

Evaluation should include a detailed medication history, assessment of treatment adherence, review of potential drug interactions, screening for alcohol and substance use, and consideration of recent medication changes. When clinically appropriate, collaboration with the treating physician to discontinue, substitute, or adjust potentially contributing medications may clarify the diagnosis and improve clinical outcomes [6,15].

In summary, comprehensive diagnostic reassessment represents an essential prerequisite for establishing a diagnosis of true treatment-resistant depression. Careful evaluation of psychiatric and medical differential diagnoses, treatment adequacy, adherence, and potentially reversible contributors helps distinguish true pharmacological resistance from pseudo-resistance. Once these factors have been systematically excluded, attention can shift toward identifying the predominant neurobiological mechanisms underlying persistent symptoms. This transition from diagnostic clarification to biological characterization provides the conceptual foundation for mechanism-based treatment selection and precision psychiatry.

## 4. Neurobiological Mechanisms Underlying Treatment-Resistant Depression

### 4.1. Monoaminergic Context

Monoaminergic models remain clinically relevant but do not account for the full heterogeneity of persistent depression. Dopaminergic and noradrenergic systems influence motivation, reward, attention, and arousal, while glutamatergic, neuroplastic, neuroendocrine, immune, metabolic, and circuit-level findings extend the biological context. Most evidence is associative and derived from groups; it does not establish discrete mechanisms in individual patients [1,6,40,43,44,94].

Although serotonergic neurotransmission remains relevant to the pathophysiology and treatment of depression, persistent depressive symptoms may also involve abnormalities in glutamatergic signaling, synaptic plasticity, stress-response systems, immune regulation, metabolic homeostasis, and genetic susceptibility [1,40,43,44,48,51,73,74,75,76,81,82,83,84,85,86,87,88]. These biological systems do not operate independently but interact dynamically through reciprocal mechanisms that influence emotional regulation, cognition, motivation, neuroplasticity, and resilience to stress.

**Figure 2:**
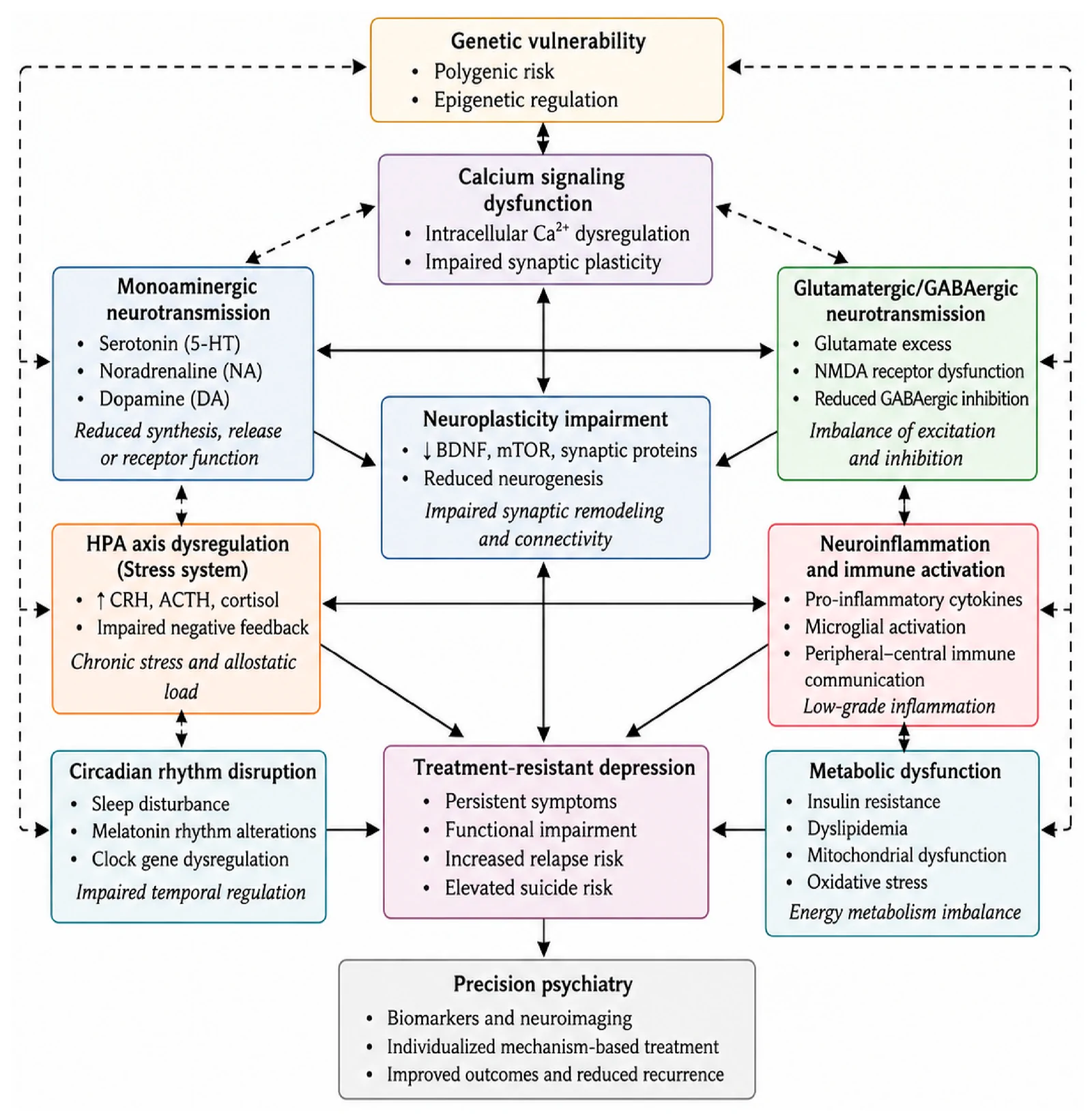
Integrated neurobiological framework for treatment-resistant depression. The figure summarizes interacting systems implicated in persistent depression; the arrows represent plausible relationships rather than established causal pathways. None of the depicted abnormalities is specific to TRD or sufficiently validated for individual diagnosis or treatment allocation.

Rather than representing competing hypotheses, these mechanisms should be viewed as complementary components of an integrated biological framework. Current evidence supports a multidimensional model in which disturbances in neurotransmission, neuroplasticity, neuroendocrine regulation, immune signaling, metabolism, and genetic vulnerability may collectively contribute to the development and persistence of TRD [1,44,94]. This integrated neurobiological framework is illustrated in Figure 2.

#### Dopaminergic and Noradrenergic Dysfunction

Although serotonergic dysfunction has traditionally been considered central to the pathophysiology of major depressive disorder, increasing evidence suggests that abnormalities in dopaminergic and noradrenergic neurotransmission also contribute to the clinical heterogeneity of treatment-resistant depression (TRD). These catecholaminergic systems regulate motivation, reward processing, attention, arousal, and executive functioning, domains that frequently remain impaired despite adequate treatment with serotonergic antidepressants. This broader neurobiological perspective may help explain why symptomatic improvement in mood does not necessarily translate into complete functional recovery in many patients with TRD [1,6,40,44,94].

Among the most prominent residual symptoms of TRD are anhedonia, reduced motivation, impaired concentration, cognitive slowing, and diminished goal-directed behaviour. These symptoms are consistent with disturbances in dopaminergic pathways involved in reward processing and motivational drive, as well as noradrenergic circuits that regulate attention, vigilance, and cognitive flexibility. Rather than representing isolated deficits, alterations in catecholaminergic neurotransmission appear to interact with multiple biological systems implicated in depression, including glutamatergic neurotransmission, neuroplasticity, stress-related neuroendocrine pathways, and inflammatory processes [1,40,43,44].

Experimental and clinical evidence also indicates that catecholaminergic neurotransmission is closely linked to mechanisms of synaptic plasticity and stress adaptation. Chronic stress, neuroendocrine dysregulation, and inflammatory activation may adversely influence dopaminergic and noradrenergic function, thereby contributing to persistent motivational deficits and impaired cognitive performance. Conversely, treatments that improve depressive symptoms may restore broader neural network function rather than acting solely through serotonergic mechanisms. These observations support an integrated neurobiological model in which catecholaminergic dysfunction represents one component of a complex network involving glutamatergic signaling, neuroplasticity, immune regulation, and stress-related pathways [40,43,44,51].

From a clinical perspective, these findings highlight the importance of recognising the multidimensional nature of TRD. Persistent symptoms such as anhedonia, reduced motivation, impaired executive functioning, and cognitive dysfunction may reflect disturbances extending beyond serotonergic neurotransmission. Appreciating this biological heterogeneity provides a rationale for individualized treatment strategies targeting multiple neurobiological pathways rather than relying exclusively on serotonergic modulation [1,6,12,40,44,94].

### 4.2. Glutamatergic and GABAergic Signaling

Although monoaminergic neurotransmission has long dominated biological models of depression, growing evidence suggests that glutamatergic signaling also plays an important role in the pathophysiology of treatment-resistant depression (TRD). This concept has gained considerable attention following the demonstration that ketamine and esketamine produce rapid antidepressant effects in patients who have not responded to conventional monoaminergic antidepressants. These observations have expanded current understanding of TRD by highlighting mechanisms beyond serotonin and noradrenaline [1,6,40,44].

The rapid antidepressant effects of ketamine have fundamentally changed contemporary concepts of TRD. Unlike conventional antidepressants, which typically require several weeks before clinical improvement becomes evident, ketamine may produce symptom improvement within hours or days in selected patients. Current evidence suggests that its antidepressant effects are mediated through modulation of glutamatergic neurotransmission, ultimately promoting synaptic recovery and functional restoration of neural circuits involved in mood regulation. These findings support the view that impaired synaptic communication, rather than monoaminergic deficiency alone, contributes to treatment resistance [1,6,40].

GABAergic neurotransmission also contributes to maintaining normal neuronal network stability by balancing excitatory glutamatergic activity. Although the precise role of GABAergic dysfunction in TRD remains incompletely understood, alterations in excitatory–inhibitory balance may influence emotional regulation, cognitive functioning, and stress responsiveness. Current evidence suggests that glutamatergic and GABAergic systems should be considered within an integrated framework involving neuroplasticity, neuroendocrine regulation, immune signaling, and metabolic function rather than as isolated neurotransmitter systems [40,43,44].

### 4.3. Neuroplasticity and Synaptic Dysfunction

Neuroplasticity refers to the brain’s capacity to adapt structurally and functionally in response to internal and external stimuli. Increasing evidence suggests that impaired neuroplasticity represents an important mechanism contributing to the persistence of treatment-resistant depression (TRD). Rather than reflecting abnormalities in a single neurotransmitter system, TRD appears to involve alterations in neuronal connectivity, synaptic function, and large-scale brain network organization, which may reduce the capacity for adaptive emotional and cognitive processing [1,40,44,77,78,79,80].

Structural and functional neuroimaging studies have consistently demonstrated abnormalities within brain regions involved in emotional regulation, cognitive control, and self-referential processing in major depressive disorder. Altered connectivity involving prefrontal, limbic, and cingulate networks has been associated with symptom severity and may influence treatment response. Although no single neuroimaging biomarker has yet demonstrated sufficient accuracy for routine clinical practice, converging evidence supports the concept that depression is associated with widespread disturbances in neural network organization rather than isolated abnormalities in specific brain regions [77,78,79,80].

Neuroplasticity is closely linked to several of the biological systems discussed throughout this review. Chronic stress, inflammatory activation, glutamatergic dysregulation, and metabolic disturbances may all adversely influence neuronal connectivity and adaptive brain function. Conversely, effective pharmacological and neuromodulatory interventions may promote functional reorganization of neural circuits, highlighting neuroplasticity as a potential final common pathway through which diverse therapeutic approaches exert their clinical effects [1,40,43,44].

### 4.4. HPA Axis and Stress Dysregulation

Chronic stress and HPA-axis regulation are associated with depression, but elevated cortisol is not a typical or defining marker of TRD. Findings vary by depressive phenotype, illness phase, circadian timing, sleep, obesity, medication, and recent stress. The evidence supports heterogeneity rather than a single hypercortisolemic TRD subtype [1,44,51].

The HPA axis is activated through the release of corticotropin-releasing hormone (CRH) from the hypothalamus, followed by adrenocorticotropic hormone (ACTH) secretion from the anterior pituitary and cortisol release from the adrenal cortex. Under physiological conditions, cortisol exerts negative feedback on both the hypothalamus and pituitary gland, thereby limiting further hormone secretion. Disturbances in this regulatory system have been reported in subsets of patients with major depressive disorder, although considerable heterogeneity exists regarding the direction and magnitude of cortisol abnormalities [44,51].

Measurement also matters. Serum, salivary, and urinary free cortisol assess different aspects of HPA function, and dynamic suppression or stimulation tests are not interchangeable with a single basal sample. A single cortisol concentration cannot diagnose HPA-axis dysregulation or allocate antidepressant treatment [1,44,51,94].

Dysregulation of the HPA axis provides a plausible biological framework linking chronic stress with alterations in brain function, immune signaling, and metabolic homeostasis. Although HPA-axis dysfunction alone is unlikely to explain the complexity of TRD, it appears to interact closely with the glutamatergic, neuroplastic, and inflammatory mechanisms discussed in the preceding sections, supporting a multidimensional model of treatment resistance [1,43,44,51].

### 4.5. Neuroinflammation

Increasing evidence suggests that immune dysregulation contributes to the pathophysiology of treatment-resistant depression (TRD) in at least a subset of patients. Rather than representing a primary inflammatory disorder, TRD appears to involve complex bidirectional interactions between the immune system, neuroendocrine regulation, neurotransmission, and neuroplasticity. Chronic psychological stress, metabolic disturbances, obesity, autoimmune diseases, and persistent low-grade systemic inflammation may all influence brain function through shared inflammatory pathways, thereby contributing to symptom persistence and reduced responsiveness to conventional antidepressant treatment [1,43,44,73,74,75,76].

Among the most consistently reported findings is the presence of elevated circulating inflammatory biomarkers, including interleukin (IL)-6, tumor necrosis factor-α (TNF-α), and C-reactive protein (CRP), in subsets of patients with major depressive disorder. Meta-analyses indicate that these abnormalities are more frequently observed in individuals with severe or treatment-resistant depression, although substantial heterogeneity exists across studies. Importantly, inflammatory biomarkers show considerable overlap between patients and healthy individuals and therefore currently lack sufficient diagnostic accuracy for routine clinical use. Nevertheless, these findings support the concept that immune activation contributes to depressive symptoms in biologically susceptible individuals [43,73,74,75,76].

Microglia, the resident immune cells of the central nervous system, have emerged as important mediators linking peripheral inflammation with alterations in brain function. Under physiological conditions, microglia contribute to immune surveillance and maintenance of neural homeostasis. Persistent activation, however, may alter synaptic function, neurotransmitter regulation, and immune signaling within the central nervous system, thereby contributing to functional changes in neural circuits involved in mood regulation. Although the precise mechanisms remain incompletely understood, experimental and clinical evidence supports a role for neuroimmune activation in the persistence of depressive symptoms in susceptible individuals [43,44,73,74].

Despite these advances, important uncertainties remain. Elevated inflammatory markers are neither universal nor specific to TRD, and considerable interindividual variability exists regarding the magnitude and clinical significance of immune activation. Current evidence therefore supports the existence of an inflammatory subtype of depression rather than a generalized inflammatory model applicable to all patients. Identification of biologically defined patient subgroups may ultimately facilitate more personalized treatment strategies, including the selective use of immunomodulatory interventions. However, further longitudinal and mechanistic studies are required before inflammatory biomarkers can be incorporated into routine clinical decision-making [73,74,75,76,94].

Neuroimmune dysregulation provides a biologically plausible mechanism linking chronic stress, metabolic abnormalities, and peripheral inflammation with altered brain function in TRD. Through interactions involving inflammatory mediators, neuroendocrine regulation, neurotransmission, and neural network function, immune dysregulation may contribute to symptom persistence in susceptible individuals. These observations further support the concept that TRD is a biologically heterogeneous disorder in which immune dysregulation represents one of several interacting pathophysiological mechanisms rather than a universal disease process [1,43,44,73,74,75,76,94].

### 4.6. Circadian Rhythm and Sleep

Disturbances in circadian rhythm and sleep are among the most common clinical features of major depressive disorder and appear to be particularly prevalent in treatment-resistant depression (TRD). Although sleep disturbances have traditionally been regarded as symptoms of depression, growing evidence suggests that they may also contribute to the development, persistence, and recurrence of depressive illness. Sleep and circadian dysfunction influence multiple biological systems implicated in TRD, including neuroendocrine regulation, emotional processing, cognitive function, and overall brain network activity [1,6,41,42,44].

Sleep disturbances in TRD are heterogeneous and may include insomnia, fragmented sleep, early morning awakening, hypersomnia, or disrupted sleep continuity. Persistent sleep impairment has consistently been associated with greater symptom severity, impaired cognitive functioning, poorer quality of life, and an increased risk of relapse following treatment. Consequently, assessment of sleep should be considered an integral component of the clinical evaluation of patients with TRD [6,41,42,44].

The relationship between sleep and depression is bidirectional. Depressive symptoms may disrupt normal sleep architecture, while chronic sleep disturbances may exacerbate emotional dysregulation, impair cognitive performance, increase stress sensitivity, and reduce treatment responsiveness. These reciprocal interactions highlight the importance of considering sleep not merely as a secondary symptom but as an important biological and clinical component of treatment-resistant depression [1,41,42,44].

Current treatment guidelines emphasize that identification and management of coexisting sleep disorders may improve overall clinical outcomes. Non-pharmacological interventions, including cognitive behavioural therapy for insomnia (CBT-I), optimization of sleep hygiene, and appropriate management of comorbid sleep disorders, should be considered alongside pharmacological treatment when clinically indicated. Recognition of sleep disturbances may therefore contribute to a more comprehensive and individualized approach to the management of TRD [6,41,42].

### 4.7. Metabolic Dysfunction

Metabolic dysfunction has emerged as an important contributor to the biological heterogeneity of treatment-resistant depression (TRD). Increasing evidence indicates that depression is frequently accompanied by metabolic abnormalities, including insulin resistance, obesity, and components of the metabolic syndrome. Rather than representing independent comorbidities, these metabolic disturbances appear to interact with neuroendocrine, inflammatory, and neurobiological pathways implicated in the persistence of depressive symptoms and reduced treatment responsiveness [1,6,43,44,48].

Among the metabolic abnormalities most consistently associated with depression is insulin resistance. A large systematic review and meta-analysis demonstrated a significant association between depression and impaired insulin sensitivity, suggesting that metabolic dysregulation may contribute to both the development and maintenance of depressive illness. Altered glucose metabolism may influence brain function through complex interactions involving inflammation, hormonal regulation, and neuronal signaling, thereby contributing to the biological heterogeneity observed in TRD [48].

Metabolic syndrome, characterized by central obesity, hypertension, dyslipidaemia, and impaired glucose regulation, is also more common among individuals with major depressive disorder than in the general population. These metabolic abnormalities are associated with increased systemic inflammation, endocrine dysregulation, and cardiovascular risk, all of which may adversely influence brain function and clinical outcomes. Although the direction of causality remains incompletely understood, current evidence suggests that depression and metabolic dysfunction reinforce one another through multiple interacting biological pathways [43,44,48,50].

From a clinical perspective, recognition of metabolic dysfunction has important implications for the assessment and management of TRD. Evaluation of metabolic risk factors, including obesity, glucose dysregulation, and other features of metabolic syndrome, may help identify potentially modifiable contributors to persistent depressive symptoms. Current evidence also supports the importance of addressing lifestyle factors, cardiovascular risk, and metabolic comorbidity as part of a comprehensive and individualized treatment approach [6,44,48,50,94].

### 4.8. Genetics, Pharmacogenomics, and Precision Psychiatry

Heritability is a population-level estimate and does not imply accurate prediction for an individual patient. Common risk variants identified through genome-wide association studies usually have small effects; rare variants, copy-number changes, de novo variation, gene–environment interaction, and epigenetic regulation may also contribute, but none currently defines TRD in routine practice. A risk association is not equivalent to a diagnostic or treatment-predictive biomarker [1,44,83,86,94].

Pharmacogenomics requires a distinction between pharmacokinetic genes, especially CYP2D6 and CYP2C19, and less consistently supported pharmacodynamic markers involving transporters, receptors, or glutamatergic pathways. Genotype may inform expected metabolism, but actual exposure also depends on phenoconversion caused by inhibitors or inducers, age, hepatic function, adherence, and polypharmacy. Commercial panels differ in included variants, algorithms, and recommendations; modest group-level improvements in some trials do not justify routine testing for all patients with TRD [81,82,83,84,85,86,87,88].

Personalized medicine in TRD extends beyond genomic testing. Current decisions can already integrate diagnosis, longitudinal course, symptom profile, functioning, previous benefit and adverse effects, comorbidity, exposure, patient goals, and treatment burden. Biological or digital measures add clinical value only if they improve prediction, decisions, or patient-important outcomes beyond this information [75,77,78,79,80,86,87,88,94].

Genetic and precision medicine approaches reinforce the concept that TRD is a biologically heterogeneous disorder arising from multiple interacting mechanisms rather than a single pathophysiological pathway. Although current genetic biomarkers have limited clinical utility when considered in isolation, continued integration of genomic, biological, and clinical information may contribute to more individualized assessment and treatment strategies in the future [1,81,82,83,84,85,86,87,88,94].

## 5. Treatment Strategies

### 5.1. General Principles of TRD Management

Management of treatment-resistant depression (TRD) should begin with confirmation that the patient has true treatment resistance rather than pseudo-resistance arising from diagnostic error, inadequate treatment exposure, poor adherence, or untreated psychiatric or medical comorbidity. Before progressing to augmentation, combination therapy, ketamine, or neuromodulation, clinicians should reassess the diagnosis, longitudinal illness course, previous treatment history, and factors that may have contributed to apparent non-response. Particular attention should be paid to previously unrecognized bipolar spectrum disorder, neurodevelopmental conditions, trauma-related disorders, personality pathology, substance use, sleep disorders, and potentially reversible medical conditions [1,6,12,15].

Each antidepressant trial should be reconstructed from prescribed and actually taken dose, time at a therapeutic dose, adherence, tolerability, interactions, and standardized symptom ratings where available. Non-response, partial response, response, remission, residual symptoms, and later loss of response are clinically different outcomes. A useful convention is that early improvement reflects at least a 20% reduction in symptom score, response at least 50%, and remission a score within the non-depressed range; thresholds remain scale-specific [1,2,6,12,15,17].

Treatment adherence should be explored in a non-judgmental manner. Non-adherence may reflect adverse effects, complex dosing regimens, limited understanding of treatment goals, cognitive or executive dysfunction, concerns regarding dependency, stigma, financial barriers, or discouragement following repeated treatment failures. Simplifying medication regimens, addressing adverse effects, providing clear psychoeducation, and involving patients actively in treatment planning may improve adherence and reduce the likelihood that modifiable treatment barriers are mistaken for biological resistance [1,6,15].

Shared decision-making is particularly important in TRD because available interventions differ substantially in efficacy, tolerability, treatment burden, monitoring requirements, accessibility, and patient acceptability. Treatment selection should therefore consider previous response patterns, symptom profile, comorbidity, suicide risk, medical status, potential adverse effects, patient preference, and practical feasibility. Repeated treatment failure may reduce hope and engagement; consequently, realistic discussion of expected benefits, uncertainties, and alternative options is essential for maintaining a collaborative therapeutic relationship [1,6,15].

Effective management requires staged reassessment and simultaneous attention to urgent depression, suicide risk, and clinically important comorbidity. The aim is not to postpone depression treatment until every coexisting condition has resolved. Instead, diagnosis, treatment adequacy, modifiable maintaining factors, and patient priorities are integrated into an explicit personalized plan, with symptom and functional outcomes measured over time [1,6,15].

#### Selecting Switching, Augmentation, or Urgent Intervention

The next step should be linked to the observed outcome rather than to trial count alone. Table 2 separates absent early improvement, partial response, residual symptoms after response, loss of a previously achieved response, urgent severe presentations, and bipolar depression. These categories are decision prompts rather than rigid rules; treatment history, tolerability, patient preference, access, metabolic vulnerability, and suicide risk remain decisive [1,6,15,18,57,58,59,60,61,62,63,64,65,66,67,68,69,70].

### 5.2. Pharmacological Augmentation Strategies

When optimization of antidepressant treatment fails to achieve adequate clinical improvement, pharmacological augmentation represents one of the most evidence-based approaches for managing treatment-resistant depression (TRD). Unlike switching antidepressants, augmentation aims to enhance the effect of an existing antidepressant through agents acting via complementary neurobiological mechanisms. Treatment selection should be individualized according to symptom profile, previous treatment response, psychiatric and medical comorbidity, suicide risk, tolerability, and patient preference. Contemporary clinical guidelines consistently recommend augmentation before repeated sequential antidepressant switching in patients who have demonstrated at least partial response to initial treatment [1,6,12].

#### 5.2.1. Lithium Augmentation

Lithium remains an established augmentation option in TRD and is especially relevant when suicide risk is prominent, provided renal, thyroid, interaction, pregnancy, and toxicity risks are addressed. In a meta-analysis of long-term randomized trials across mood disorders, lithium reduced suicide versus placebo (odds ratio 0.13, 95% CI 0.03–0.66); the unipolar-depression subgroup estimate was 0.36 (95% CI 0.13–0.98). These estimates support a clinically important antisuicidal signal but derive from relatively rare events and should not replace individualized risk management [59,63,68].

#### 5.2.2. Second-Generation Antipsychotics

Several second-generation antipsychotics have demonstrated efficacy as augmentation in antidepressant-resistant unipolar major depression. A meta-analysis of 16 placebo-controlled trials (*n* = 3480) found higher response (odds ratio 1.69, 95% CI 1.46–1.95) and remission (odds ratio 2.00, 95% CI 1.69–2.37), but discontinuation because of adverse events was also increased (odds ratio 3.91, 95% CI 2.68–5.72). Aripiprazole, quetiapine extended-release, brexpiprazole, and olanzapine–fluoxetine have the most established evidence or regulatory support in relevant settings. The modest average benefit must be balanced against akathisia, sedation, weight gain, dyslipidaemia, glucose dysregulation, and patient preference; baseline and follow-up metabolic monitoring are therefore essential [63,64,65,66,67].

#### 5.2.3. Thyroid Hormone Augmentation

Triiodothyronine (T3) augmentation has been used for several decades as an adjunctive treatment for patients with inadequate antidepressant response, even in the absence of overt thyroid disease. Clinical studies suggest that T3 may accelerate antidepressant response and improve remission rates in selected patients, although the evidence base is smaller than for lithium or atypical antipsychotics. Potential mechanisms include modulation of monoaminergic neurotransmission, enhancement of neuroplasticity, and effects on cerebral metabolism. Before initiation, thyroid function should be assessed, and treatment should be accompanied by appropriate endocrine monitoring, particularly in patients with cardiovascular disease or osteoporosis risk [6,12,15].

#### 5.2.4. Combination Antidepressant Therapy

For patients with inadequate response to monotherapy, combining antidepressants with complementary pharmacological mechanisms represents another evidence-based strategy. Common combinations include an SSRI or SNRI with mirtazapine or bupropion, aiming to target broader neurochemical pathways than single-agent therapy alone. Although combination treatment may benefit selected individuals, evidence suggests that it is not universally superior to well-conducted augmentation strategies and may increase the risk of adverse effects, drug interactions, and treatment complexity. Careful consideration should therefore be given to previous treatment history, tolerability, and the potential for serotonin syndrome when combining serotonergic agents [6,63,65].

Pharmacological augmentation should be regarded as an individualized, mechanism-informed strategy rather than a uniform algorithm. The choice of augmentation depends on clinical presentation, biological characteristics, previous treatment response, comorbidities, safety considerations, and patient preferences. As the understanding of TRD increasingly shifts toward biological heterogeneity, future advances in biomarkers and precision psychiatry may further improve selection of augmentation strategies most likely to benefit individual patients.

### 5.3. Ketamine and Esketamine

The introduction of ketamine and its S-enantiomer esketamine has fundamentally changed the therapeutic landscape of treatment-resistant depression (TRD). Unlike conventional antidepressants, which primarily target monoaminergic neurotransmission and often require several weeks before clinical improvement becomes evident, ketamine produces rapid antidepressant effects through modulation of glutamatergic neurotransmission. This novel mechanism has provided important support for the concept that disrupted glutamate signaling and impaired synaptic plasticity contribute to the pathophysiology of TRD. Consequently, ketamine-based therapies have become valuable treatment options for carefully selected patients with severe depression, particularly when rapid symptom reduction is clinically necessary [40,60,61].

NMDA-receptor antagonism, altered AMPA throughput, BDNF- and mTOR-related signaling, and changes in synaptic function form a useful mechanistic model of ketamine action. The sequence is not a complete or clinically validated explanation of response, however, and peripheral BDNF or related measures cannot be used to infer synaptogenesis or select an individual patient for treatment [40].

One of the most distinctive features of ketamine is the rapid reduction in depressive symptoms, often occurring within hours after administration and substantially earlier than with conventional antidepressants. Randomized controlled trials and meta-analyses have consistently demonstrated clinically meaningful improvements in depressive symptoms among patients with TRD following intravenous ketamine or intranasal esketamine. However, the antidepressant effects are often transient after a single administration, necessitating repeated treatment sessions or maintenance strategies to sustain remission. Long-term treatment protocols continue to evolve as additional safety and effectiveness data become available [40,60,61].

Ketamine and esketamine can reduce suicidal ideation rapidly in selected patients, but improvement in thoughts is not evidence of reduced suicide attempts or mortality. They should not be described as stand-alone suicide-prevention treatments. Comprehensive assessment, safety planning, monitoring, and treatment of the underlying disorder remain necessary [40,60,61].

Appropriate patient selection is essential to maximize benefit while minimizing potential risks. Ketamine and esketamine are generally reserved for adults with confirmed treatment-resistant depression who have failed to respond adequately to multiple evidence-based antidepressant treatments. Before initiation, clinicians should reassess the diagnosis, confirm treatment adequacy, evaluate psychiatric and medical comorbidities, and exclude alternative explanations for persistent symptoms. Particular caution is warranted in patients with psychotic disorders, uncontrolled hypertension, significant cardiovascular disease, active substance use disorders, or a history of ketamine misuse. Because transient increases in blood pressure, dissociative symptoms, dizziness, nausea, and perceptual disturbances may occur, treatment should be administered within structured clinical settings with appropriate monitoring [40].

Despite their substantial therapeutic potential, ketamine and esketamine have several limitations. Their relatively short duration of action frequently necessitates repeated administration, and optimal long-term maintenance strategies remain incompletely defined. Important questions also remain regarding durability of response, predictors of treatment success, long-term cognitive safety, abuse liability, and cost-effectiveness. Furthermore, only a subset of patients achieve sustained remission, highlighting the biological heterogeneity of TRD. Ongoing research aimed at identifying clinical, neurobiological, and molecular biomarkers may improve patient selection and facilitate more personalized use of glutamatergic therapies. Future studies combining ketamine treatment with biomarker-guided precision psychiatry may further enhance long-term outcomes in treatment-resistant depression [40].

Ketamine and esketamine represent major advances in the treatment of TRD by providing rapid symptom relief through mechanisms distinct from conventional monoaminergic antidepressants. Although they have significantly expanded available therapeutic options, careful patient selection, structured monitoring, and integration within a comprehensive long-term treatment plan remain essential for achieving sustained clinical benefit.

### 5.4. Neuromodulation

Neuromodulation has become an increasingly important component of the therapeutic armamentarium for treatment-resistant depression (TRD), particularly in patients who fail to respond to multiple pharmacological interventions or who require rapid symptom improvement because of severe illness or suicidality. Unlike pharmacological treatments, neuromodulation directly targets dysfunctional neural circuits implicated in mood regulation and cognitive control. Available techniques differ substantially in invasiveness, efficacy, durability of response, adverse-effect profiles, and level of supporting evidence. Current international guidelines recommend selecting neuromodulatory interventions according to illness severity, treatment history, medical comorbidity, patient preference, and local availability [6,69,70,71,72].

#### 5.4.1. Electroconvulsive Therapy (ECT)

Electroconvulsive therapy (ECT) remains the most effective acute treatment for severe treatment-resistant depression and continues to represent the gold standard for patients with severe, psychotic, catatonic, or life-threatening depressive episodes. Response rates frequently exceed those achieved with pharmacological therapies, particularly in patients with marked psychomotor retardation, psychotic features, severe malnutrition, or persistent suicidal risk.

The therapeutic mechanisms of ECT are not fully understood but appear to involve widespread modulation of functional brain networks, enhancement of neuroplasticity, increased expression of brain-derived neurotrophic factor (BDNF), normalization of hypothalamic–pituitary–adrenal axis activity, and restoration of connectivity within fronto-limbic circuits. Although transient cognitive adverse effects, including temporary memory impairment and confusion, may occur, modern ECT techniques using individualized stimulus dosing and unilateral electrode placement have substantially improved tolerability. Maintenance pharmacotherapy or continuation ECT is often required to reduce relapse following successful acute treatment [62,69].

#### 5.4.2. Repetitive Transcranial Magnetic Stimulation (rTMS)

Repetitive transcranial magnetic stimulation (rTMS) is a non-invasive neuromodulation technique that uses magnetic pulses to modulate cortical excitability, most commonly targeting the left dorsolateral prefrontal cortex. Numerous randomized controlled trials and meta-analyses have demonstrated significant antidepressant efficacy in patients with TRD, although response rates are generally lower than those observed with ECT.

Compared with ECT, rTMS offers important practical advantages, including the absence of general anesthesia, minimal cognitive adverse effects, and excellent overall tolerability. Accelerated protocols and intermittent theta burst stimulation (iTBS) have further reduced treatment duration while maintaining comparable efficacy. rTMS is particularly attractive for patients with moderate treatment resistance who prefer non-pharmacological treatment or who are unable to tolerate medication-related adverse effects [69,70].

#### 5.4.3. Vagus Nerve Stimulation (VNS)

Vagus nerve stimulation (VNS) involves chronic electrical stimulation of the left cervical vagus nerve through an implanted pulse generator. Although antidepressant effects typically develop gradually over several months, long-term observational studies suggest that VNS may provide sustained symptom improvement in carefully selected patients with chronic and highly treatment-resistant depression.

The proposed mechanisms include modulation of monoaminergic neurotransmission, autonomic regulation, inflammatory pathways, and large-scale neural networks involved in emotional regulation. Because implantation requires surgery and treatment costs remain substantial, VNS is generally reserved for patients with severe chronic TRD who have failed multiple pharmacological and neuromodulatory interventions [69,71].

#### 5.4.4. Deep Brain Stimulation (DBS)

Deep brain stimulation (DBS) represents the most invasive neuromodulation strategy currently under investigation for severe refractory depression. Electrodes are surgically implanted into specific brain regions implicated in mood regulation, including the subcallosal cingulate cortex, ventral capsule/ventral striatum, nucleus accumbens, and medial forebrain bundle.

Initial open-label studies demonstrated encouraging clinical responses; however, randomized controlled trials have produced mixed results, reflecting the biological heterogeneity of TRD and challenges in identifying optimal stimulation targets and patient populations. Consequently, DBS remains an investigational treatment and is generally limited to specialized research centres or highly selected patients with extreme treatment resistance after failure of established therapeutic options [69,72].

Neuromodulation provides effective treatment alternatives for patients with TRD who fail to respond adequately to pharmacological therapies. ECT remains the intervention with the strongest evidence and highest remission rates, whereas rTMS offers an effective non-invasive option with excellent tolerability. VNS may provide long-term benefit in carefully selected patients with chronic illness, while DBS continues to evolve as an experimental precision-based intervention. Future advances integrating neuroimaging, electrophysiology, and biomarker-guided patient selection may further improve the effectiveness and personalization of neuromodulation therapies in TRD [6,62,69,70,71,72].

### 5.5. Psychological, Sleep, and Medical Adjuncts

#### 5.5.1. Psychological Maintaining Processes and Apparent Treatment Resistance

Psychological treatment is part of core TRD care rather than an optional intervention after pharmacotherapy has failed. Rumination can repeatedly reactivate negative self-referential material and displace problem solving; behavioral and experiential avoidance can provide short-term relief while preventing corrective learning and reducing rewarding activity; anhedonia and inactivity can progressively narrow positive reinforcement; insomnia and circadian disruption can amplify affective reactivity, fatigue, and cognitive inefficiency; and interpersonal conflict, chronic stress, trauma-related threat processing, hopeless expectations, and emotion dysregulation can maintain symptoms between medication visits. Executive dysfunction or neurodevelopmental difficulties may also impair planning, attendance, homework completion, adherence, and implementation of coping strategies. Persistent symptoms driven by these processes can appear pharmacologically resistant even when the principal unmet need is formulation-based psychological, sleep, social, or functional intervention [6,9,11,15,28,30,34,35,36,37,38,95].

Psychotherapy nonresponse should itself be reassessed rather than treated as evidence of global psychological refractoriness. Relevant questions include whether the previous intervention targeted the active maintaining process; whether session number, intensity, attendance, between-session practice, and therapeutic alliance were adequate; whether severe cognitive symptoms, active trauma, personality-related difficulties, autism, ADHD, substance use, poverty, caregiving demands, or unstable housing impeded engagement; and whether treatment was adapted to communication, sensory, executive, and motivational needs. A failure of one manualized therapy under inadequate or mismatched conditions does not establish resistance to psychotherapy as a class.

Evidence supports psychotherapy as an active treatment component in TRD, although the evidence base is smaller than for several biological interventions. A Cochrane review of six randomized trials (*n* = 698) found that psychotherapy added to usual antidepressant care improved short-term self-reported symptoms (standardized mean difference −0.40, 95% CI −0.65 to −0.14; *n* = 635), response (risk ratio 1.80, 95% CI 1.20–2.70; *n* = 556), and remission (risk ratio 1.92, 95% CI 1.46–2.52; *n* = 635), without a clear difference in short-term dropout (risk ratio 0.85, 95% CI 0.58–1.24; *n* = 698). Most evidence was driven by adjunctive cognitive behavioral therapy and one large trial, so precision across specific psychotherapy types and patient subgroups remains limited [95].

#### 5.5.2. Functional Recovery and Personalized Psychological Care

Symptom change and functional recovery are related but non-equivalent outcomes. Work or study capacity, relationships, self-care, cognitive efficiency, anhedonia, sleep, quality of life, safety, treatment burden, relapse, and progress toward patient-defined goals should be measured separately. A patient may meet a symptom-response threshold yet remain unable to resume valued roles, while another may achieve meaningful functional gains without full scale-defined remission. Such divergence should guide the next intervention rather than automatically trigger another medication trial [1,6,15].

Treatment should be matched to the formulation and the outcome target. Cognitive and behavioral approaches may address rumination, avoidance, inactivity, hopeless predictions, and relapse prevention; behavioral activation can directly increase contact with reinforcement; insomnia-focused treatment can reduce a potent maintaining factor; trauma-focused treatment is appropriate when trauma-related psychopathology is active; and longer-term or interpersonal approaches may be required for enduring relational or personality-related difficulties. Neurodevelopmental adaptations may include greater structure, explicit goals, shorter steps, written prompts, sensory or communication adjustments, and practical support for implementation. Progress should be reviewed with both symptom and functional measures, and the formulation should be revised when the expected mechanism of change is not observed [9,11,15,28,30,34,35,36,37,38,95]. Selected quantitative estimates supporting the clinical interpretation are summarized in Table 3.

### 5.6. Personalized and Mechanism-Based Treatment

A personalized medicine approach to TRD does not require a validated biological subtype before care can be individualized. Its immediate task is to match established interventions to the verified diagnosis, severity, urgency, previous response, adverse-effect vulnerability, comorbidity, functional goals, treatment burden, and patient preference. Biological stratification remains a research aim rather than a prerequisite for good personalized care [1,73,74,75,76,77,78,79,80,81,82,83,84,85,86,87,88,94].

**Figure 3:**
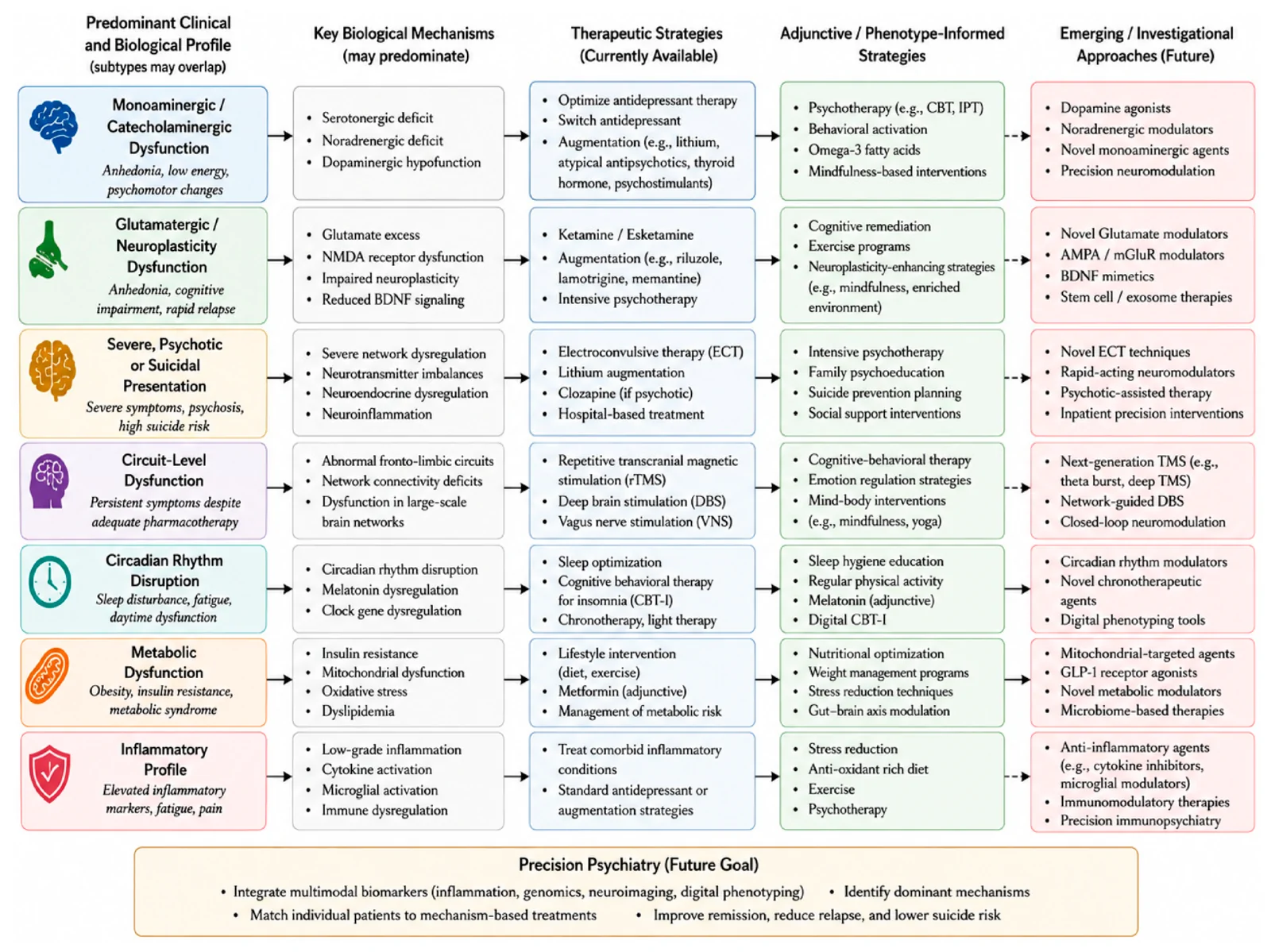
Proposed framework linking clinical phenotype, candidate mechanisms, and treatment selection in treatment-resistant depression. Established interventions remain guided primarily by diagnosis, severity, urgency, previous treatment, comorbidity, safety, and patient preference. The phenotype–mechanism links shown here are research hypotheses and should not be interpreted as a validated treatment algorithm.

Group-level differences have been reported in inflammatory, glutamatergic, neuroendocrine, circadian, metabolic, neuroplastic, and circuit measures. These associations are heterogeneous and do not show that a dominant mechanism has been identified in a particular patient. They generate hypotheses for stratified trials; they do not presently justify direct biomarker-to-treatment matching [43,48,73,74,75,76,77,78,79,80,94]. The relationship between clinical phenotype, candidate mechanisms, and treatment selection is illustrated in Figure 3.

No inflammatory, neuroendocrine, metabolic, imaging, or genetic marker currently has sufficient discrimination and prospective validation to select treatment for an individual patient with TRD. Multimodal panels may outperform single markers, but their incremental clinical value over careful phenotyping has not been established [73,74,75,76,77,78,79,80,94].

Neuroimaging has identified group-level abnormalities in cortical structure and fronto-limbic network function and has generated candidate predictors of treatment outcome. These findings remain research tools because acquisition, analysis, thresholds, and external validation are not standardized for individual clinical decisions [77,78,79,80].

Pharmacogenomic testing can inform exposure and tolerability for selected drug–gene pairs, particularly where CYP2D6 or CYP2C19 metabolism is relevant. Evidence for improved depressive outcomes is heterogeneous, and commercial systems may disagree in their recommendations; pharmacogenomics therefore complements rather than replaces clinical assessment [81,82,83,84,85,86,87,88].

The defensible near-term role of precision psychiatry is consequently narrower than direct biomarker-to-drug matching. Its immediate contribution is to combine longitudinal treatment history, symptom profile, comorbidity, adverse-effect vulnerability, and selected biological information into explicit, testable treatment decisions [75,79,80,81,82,83,84,85,86,87,88,94].

## 6. Suicide Risk

Suicidal ideation, attempts, and mortality are more frequent in TRD cohorts, but TRD is not an isolated causal exposure. Severity, duration, previous attempts, bipolarity, personality disorder, substance use, chronic pain, social adversity, and barriers to care may confound or mediate the association. Suicide-risk assessment should therefore be repeated throughout treatment and anchored in an individualized formulation rather than the TRD label [1,6,12,52,53,54,55,56,57,58].

### 6.1. Risk Factors for Suicide

Suicide risk in TRD is multifactorial and dynamic. Previous suicide attempts remain the strongest predictor of future suicidal behaviour, while persistent suicidal ideation, severe hopelessness, recurrent depressive episodes, psychiatric hospitalization, comorbid anxiety disorders, substance use disorders, personality disorders, chronic pain, social isolation, and psychosocial adversity further increase vulnerability. Diagnostic reassessment is particularly important because previously unrecognized bipolar disorder, adult ADHD, autism spectrum disorder, personality pathology, or trauma-related disorders may contribute both to treatment resistance and to increased suicide risk. Risk should therefore be evaluated longitudinally, recognizing that suicidal intent may fluctuate considerably over time rather than remain constant [52,53,54,55,56,57,58].

### 6.2. Suicide Risk Assessment

Comprehensive suicide risk assessment extends beyond asking whether suicidal thoughts are present. Evaluation should include suicidal ideation, intent, planning, access to lethal means, previous attempts, impulsivity, psychiatric comorbidity, substance misuse, psychosocial stressors, protective factors, family support, and recent changes in mental state. Structured clinical assessment combined with professional judgement remains the recommended approach, as no single risk assessment instrument reliably predicts future suicidal behaviour at the individual level. Continuous reassessment is particularly important during periods of medication changes, clinical deterioration, hospital discharge, or major psychosocial stressors [57,58].

### 6.3. Suicide Prevention Strategies

Effective suicide prevention combines treatment of the depressive episode with an individualized safety plan, restriction of access to lethal means, involvement of supportive others when appropriate, close follow-up, crisis access, and management of comorbidity. Lithium is supported by evidence on suicidal behaviour and mortality in mood disorders. Ketamine and esketamine may reduce suicidal ideation rapidly, but this outcome must not be equated with prevention of attempts or death. ECT should be considered when severe depression requires a rapid and robust response [59,60,61,62].

### 6.4. Emerging Approaches to Suicide-Risk Prediction

Despite advances in treatment, accurate prediction of suicidal behaviour remains one of the greatest challenges in psychiatry. Current research focuses on identifying biological, neuroimaging, digital, and clinical biomarkers capable of improving individualized risk prediction and facilitating earlier intervention. Integration of biomarker research, artificial intelligence, ecological momentary assessment, and precision psychiatry may eventually enable more personalized suicide prevention strategies. Until such approaches become clinically validated, repeated clinical assessment, individualized management, and continuity of care remain the foundation of suicide prevention in patients with treatment-resistant depression [1,6,57,58].

Inflammatory and kynurenine-pathway findings offer plausible links between stress, immune signaling, and suicidal behaviour, and immunomodulatory strategies are under investigation. The evidence remains largely associative and treatment-specific evidence is preliminary; inflammatory markers should not be used to estimate individual suicide risk or select immunomodulatory treatment outside validated indications [96].

## 7. Biomarkers and Precision Psychiatry

Biomarker claims in TRD require a defined intended use. Diagnostic biomarkers identify a condition; prognostic biomarkers estimate outcome independent of a particular treatment; predictive biomarkers identify differential treatment benefit; monitoring and safety biomarkers follow disease or toxicity; pharmacodynamic biomarkers show biological response; and mechanistic biomarkers interrogate pathways. Evidence for one role cannot be transferred automatically to another. No biomarker currently has adequate validation for routine TRD diagnosis or treatment allocation [73,74,75,76,77,78,79,80,81,82,83,84,85,86,87,88,94].

### 7.1. Inflammatory Biomarkers

Meta-analytic evidence supports small group-level differences in several inflammatory markers in depression, with substantial between-study heterogeneity and overlap between patients and controls. CRP, IL-6, and TNF-α are influenced by infection, adiposity, smoking, age, medication, sleep, stress, and physical activity. An inflammatory association is neither a diagnostic marker of TRD nor proof of differential benefit from ketamine or an anti-inflammatory treatment [73,74,75,76].

### 7.2. Neuroimaging Biomarkers

Advances in structural and functional neuroimaging have improved understanding of the neural circuits involved in TRD. Functional magnetic resonance imaging (fMRI), positron emission tomography (PET), diffusion tensor imaging (DTI), and connectivity analyses have consistently demonstrated abnormalities involving fronto-limbic circuits, the anterior cingulate cortex, amygdala, hippocampus, and large-scale functional brain networks. Neuroimaging markers may eventually contribute to predicting treatment response, particularly for neuromodulation techniques such as electroconvulsive therapy and repetitive transcranial magnetic stimulation. However, methodological heterogeneity, limited reproducibility, and high cost currently restrict their application to research settings [77,78,79,80].

### 7.3. Pharmacogenomics

Pharmacogenomic testing is most defensible when a specific drug–gene interaction can clarify exposure or tolerability, especially for CYP2D6 or CYP2C19 substrates. Pharmacodynamic associations are less consistent, and commercial panels are not interchangeable. Results must be interpreted alongside phenoconversion, drug interactions, age, hepatic function, adherence, and polypharmacy. Evidence does not support routine panel testing for every patient with TRD [81,82,83,84,85,86,87,88].

### 7.4. Clinical Implications

Current evidence does not justify routine biomarker-guided treatment selection in TRD. As summarized in Table 4, a candidate test requires analytical validity, clinical validity, reproducibility, calibration, external validation, feasible thresholds, and evidence that its use improves decisions and patient-important outcomes beyond clinical assessment. Statistical association or discrimination within a single dataset is insufficient [73,74,75,76,77,78,79,80,81,82,83,84,85,86,87,88,94].

## 8. Discussion and Clinical Implications

This review supports a specific revision of the TRD construct. TRD is useful as an operational description of unsuccessful adequate treatment, but it is not an etiologically homogeneous diagnosis. Inadequate exposure, an incorrect diagnosis, clinically important comorbidity, and depressive illness maintained by different psychological, social, and biological processes require different responses and should not be collapsed into one explanation.

Taken together, the three proposed figures provide complementary conceptual frameworks illustrating diagnostic reassessment, biological heterogeneity, and mechanism-informed treatment selection. They are intended to support clinical and theoretical understanding rather than to function as validated evidence-based clinical algorithms.

The practical consequence is a staged but not needlessly delayed assessment. Confirm major depression, treatment adequacy, adherence, and exposure; determine whether another disorder better explains the episode; and then identify comorbidities and maintaining factors. Severe depression and suicide risk should be treated promptly, while ADHD, ASD, trauma-related illness, personality disorder, sleep disease, substance use, and medical conditions are addressed concurrently or sequentially according to urgency and feasibility.

The second component is a restrained use of mechanism-based reasoning. Glutamatergic signaling, plasticity, stress physiology, inflammation, circadian disruption, metabolism, and neural circuitry offer coherent explanations for heterogeneity, but current evidence does not permit clinicians to infer a dominant mechanism from symptoms or a single biomarker. Treatment choice must therefore remain evidence-based and clinically anchored, while proposed phenotype–mechanism links are tested prospectively.

The framework contributes a testable personalized medicine sequence rather than a new subtype system: verify diagnosis and treatment exposure; characterize severity, urgency, comorbidity, psychological maintaining processes, function, and patient priorities; select an intervention with established evidence and acceptable burden; and measure symptoms, anhedonia, cognition, functioning, quality of life, safety, relapse, and treatment acceptability over time. Biological measures earn a place only if they improve this sequence beyond clinical data alone.

### Strengths and Limitations

The principal strength of this review is the integration of diagnosis, psychological and functional assessment, suicide risk, neurobiology, and treatment within one clinical sequence. It also makes explicit a recurrent inferential error: association with depression is not evidence of causation, individual classification, or treatment prediction in TRD.

The conclusions are limited by the structured narrative design, the use of one bibliographic database supplemented by reference-list searching, heterogeneous definitions and populations, and the absence of de novo risk-of-bias or certainty grading. Search-hit counts overlap across domains and no PRISMA-style unique-record flow was constructed. Effect measures could not be presented in one uniform metric across clinical, biological, psychological, and treatment studies; numerical estimates were therefore reported when they were available and interpretable, with heterogeneity and adverse-event burden noted explicitly. Several mechanistic findings derive from major depression rather than rigorously defined TRD, and most proposed biomarkers lack prospective external validation. The figures and tables are therefore conceptual and operational aids, not validated treatment algorithms.

The central research question is no longer whether TRD is heterogeneous, but whether clinically defined phenotypes combined with reproducible biological measures can improve treatment allocation and outcomes. Trials should prospectively define resistance, exclude pseudo-resistance, stratify patients before treatment, and evaluate remission, functioning, relapse, adverse effects, and suicide-related outcomes.

## 9. Conclusions

Treatment-resistant depression is an operational treatment-history category containing clinically different problems. A personalized medicine assessment first confirms major depressive disorder, adequate treatment exposure and adherence, and the observed outcome. It then distinguishes diagnostic alternatives from comorbidities and modifiable psychological, social, sleep-related, substance-related, and medical factors. Depression and urgent suicide risk should be treated without waiting for all comorbid conditions to resolve.

Biological heterogeneity is supported at the group level but has not produced validated individual TRD subtypes or reliable biomarker-guided prescribing. Glutamatergic signaling, plasticity, stress regulation, inflammation, circadian function, metabolism, genetics, and neural circuitry remain interacting research domains. Current treatment selection must remain anchored in diagnosis, severity, urgency, previous response, comorbidity, functioning, safety, feasibility, patient preference, and comparative evidence.

The scientific contribution is a staged, testable personalized medicine framework that separates diagnostic correction, treatment adequacy, course modifiers, and mechanism-informed planning. Its central proposition is deliberately falsifiable: multimodal measurement should influence care only when it adds prospectively validated predictive value beyond high-quality clinical and functional assessment. Stratified trials should test that added value using remission, functioning, quality of life, relapse, adverse effects, treatment burden, and suicide-related outcomes.

## Figures and Tables

**Table 1:** Operational sequence for confirming and characterizing treatment-resistant depression.

Step	What to Establish	Practical Criterion	Interpretation
1. Diagnosis	Current MDD episode and course	Clinical diagnostic assessment; examine bipolarity, psychosis, substance/medication effects, and medical causes	Alternative primary diagnoses are not TRD
2. Trial count	At least two antidepressant trials	Trials occurred during the same current episode and are documented separately	Historical failures outside the episode add context but do not define current TRD
3. Adequacy	Dose and duration	Therapeutic or maximally tolerated dose for an adequate acute trial, commonly at least 6 weeks unless intolerance or urgent change intervenes	Subtherapeutic or prematurely stopped treatment is not an adequate failure
4. Exposure	Adherence and pharmacological exposure	Non-judgmental interview, records and dispensing history; drug concentration only when clinically useful	Insufficient exposure is pseudo-resistance
5. Outcome	Standardized symptom change and remission	Early improvement ≥20%; response ≥50%; remission within the non-depressed range, using scale-specific thresholds	Distinguish non-response, partial response, response, remission, residual symptoms, and loss of response
6. Modifiers	Comorbidity and maintaining factors	Psychiatric, psychological, sleep, substance-related, interpersonal, social, and medical assessment	May coexist with MDD and modify treatment; not automatically pseudo-resistance
7. Classification	TRD versus advanced treatment-refractory depression	TRD: failure of ≥2 adequate antidepressant trials in the same episode. Refractory: persistent nonresponse after ≥2 adequate antidepressant trials, ≥1 evidence-based augmentation strategy, and adequate ECT unless contraindicated, unavailable, or declined.	Refractory is a pragmatic descriptor of more advanced TRD, not a separate diagnosis or an interchangeable synonym; always state failed modalities and adequacy.

**Table 2:** Response-pattern and diagnosis-based framework for switching, augmentation, and urgent treatment.

Clinical Pattern	Preferred Next Step	Clinical Rationale	Safety and Diagnostic Modifiers
Absent early improvement or nonresponse (<20% symptom reduction)	Reconfirm exposure and diagnosis; optimize if inadequate, otherwise consider switching to a treatment with a different mechanism.	Continuing an ineffective strategy has limited value when verified exposure produced essentially no benefit.	Reassess bipolarity, substance/medication effects, adherence, interactions, and urgent suicide risk.
Partial response (approximately 20–49%)	Optimize dose/duration if tolerated; then preferentially consider augmentation or targeted psychotherapy while retaining the partially effective antidepressant.	Preserves an established signal of benefit while addressing residual symptom domains.	Choose the augmenter according to renal/thyroid status, weight, diabetes, dyslipidaemia, akathisia, interactions, and patient preference.
Response (≥50%) with residual symptoms or functional impairment	Target the residual domain: psychotherapy/behavioral activation, sleep treatment, cognitive or anhedonia-focused strategies, rehabilitation, or carefully selected augmentation.	Symptom response is not remission or functional recovery; residual symptoms predict relapse.	Measure functioning and quality of life separately; avoid adding treatment burden without a defined target.
Loss of a previously achieved response	Check adherence, new interactions, substance use, sleep, stressors, medical illness, and bipolar course; re-optimize the previously effective regimen or switch/augment according to the cause.	Relapse or tachyphylaxis should not automatically be coded as primary nonresponse.	Repeat suicide-risk assessment and evaluate emerging mixed/manic symptoms.
Psychotic, catatonic, severely malnourished, or immediately life-threatening depression	Prioritize urgent specialist treatment; ECT is a leading option. Ketamine/esketamine may rapidly reduce depressive symptoms or suicidal ideation in selected monitored patients.	Urgency and likelihood of rapid benefit outweigh routine stepwise sequencing.	Rapid improvement in suicidal ideation is not proof of reduced attempts or mortality; maintain safety planning and follow-up.
Bipolar depression	Use a bipolar-depression guideline and mood-stabilizing/approved bipolar strategies rather than a unipolar TRD algorithm.	Apparent unipolar resistance may reflect an incorrect diagnosis; treatment-resistant bipolar depression is a distinct clinical problem.	Avoid unopposed antidepressant escalation; consider switch risk, mixed features, metabolic burden, and suicide risk.

**Table 3:** Selected quantitative estimates used to support clinical interpretation across review domains.

Domain and Source	Population/Evidence	Effect Estimate (95% CI)	Clinical Interpretation and Heterogeneity
Psychotherapy added to usual care [95]	6 RCTs; *n* = 698 adults with antidepressant-resistant unipolar depression	Symptoms: SMD −0.40 (−0.65 to −0.14); response RR 1.80 (1.20–2.70); remission RR 1.92 (1.46–2.52)	Moderate average short-term benefit; evidence for specific therapy types and subgroups remains limited. Heterogeneity was handled with random-effects models; the abstract did not report a single I^2^ for all outcomes.
Second-generation antipsychotic augmentation [66]	16 placebo-controlled RCTs; *n* = 3480	Response OR 1.69 (1.46–1.95); remission OR 2.00 (1.69–2.37); adverse-event discontinuation OR 3.91 (2.68–5.72)	Efficacy is clinically relevant but accompanied by higher treatment burden; metabolic and neurological adverse effects must shape selection. Fixed-effect pooling was used; a single I^2^ was not reported in the abstract.
Lithium and suicide outcomes [59]	48 long-term RCTs; *n* = 6674 across mood disorders	Suicide vs. placebo OR 0.13 (0.03–0.66); unipolar subgroup OR 0.36 (0.13–0.98)	Potentially important antisuicidal benefit, but rare-event uncertainty remains. The abstract reported small statistical variation across active-comparator results rather than a single I^2^.
Autoimmune thyroiditis and depression [46]	19 studies; 21 samples; 35,168 participants in depression analyses	Depression OR 3.56 (2.14–5.94); I^2^ = 92.1%	Strong association but very high heterogeneity; this supports diagnostic awareness, not causal attribution or thyroid-based treatment of TRD.

**Table 4:** Biomarker roles, evidentiary requirements, and present clinical status in treatment-resistant depression.

Biomarker Role	Clinical Question	Required Evidence	Current Status in TRD
Diagnostic	Does the patient have TRD or another condition?	Sensitivity, specificity, reference standard, external validation	No validated biomarker
Prognostic	What outcome is likely regardless of treatment?	Calibration and discrimination in representative prospective cohorts	Candidate associations only
Predictive	Who benefits more from one treatment than another?	Replicated treatment-by-biomarker interaction in prospective trials	No routine treatment-allocation marker
Monitoring/safety	Is illness or toxicity changing?	Reliable within-person change linked to decisions and outcomes	Established only for treatment-specific safety monitoring
Pharmacodynamic	Did the intervention alter its intended target?	Validated target-engagement measure related to outcome	Primarily research use
Mechanistic	Which pathway is implicated?	Reproducible biological association with causal support	Hypothesis-generating; not an allocation tool

## Data Availability

No new data were created or analyzed in this study. Data sharing is not applicable to this article.

## Supplementary Materials

The following supporting information can be downloaded at: https://www.mdpi.com/article/10.3390/jpm16090468/s1, Table S1: Complete PubMed/MEDLINE search strategies.
