# Supplementary material for: Treatment-Resistant Depression as a Heterogeneous Clinical Syndrome: A Personalized Medicine Framework for Diagnostic Reassessment, Suicide Risk, and Mechanism-Based Treatment
Source: J Pers Med. 2026 Sep 13;16(9):468. doi: 10.3390/jpm16090468 (PMC13608669; doi:10.3390/jpm16090468)
Supplement: Supplementary file 1 [file jpm-16-00468-s001.zip › jpm-4543229-supplementary.pdf]

Supplementary Table S1

Complete PubMed/MEDLINE search strategies

**Coverage**	Database inception to 31 July 2026
**Revision rerun**	10 September 2026, retaining the 31 July 2026 publication-date cutoff
**Database**	PubMed/MEDLINE

Common Search Components

TRD block: ("treatment-resistant depression"[Title/Abstract] OR "treatment refractory depression"[Title/Abstract] OR "refractory depression"[Title/Abstract] OR "Depressive Disorder, Treatment-Resistant"[Mesh])

Date block: ("1900/01/01"[Date - Publication] : "2026/07/31"[Date - Publication])

Complete Domain-Specific Strategies and Record Counts

Definitions and diagnostic reassessment

Records returned: **603**

```
("treatment-resistant depression"[Title/Abstract] OR "treatment refractory depression"[Title/Abstract] OR "refractory depression"[Title/Abstract] OR "Depressive Disorder, Treatment-Resistant"[Mesh]) AND (definition[Title/Abstract] OR staging[Title/Abstract] OR "pseudo-resistance"[Title/Abstract] OR reassessment[Title/Abstract] OR diagnosis[Title/Abstract]) AND ("1900/01/01"[Date - Publication] : "2026/07/31"[Date - Publication])
```

Differential diagnosis and modifiers

Records returned: **1,342**

```
("treatment-resistant depression"[Title/Abstract] OR "treatment refractory depression"[Title/Abstract] OR "refractory depression"[Title/Abstract] OR "Depressive Disorder, Treatment-Resistant"[Mesh]) AND (bipolar[Title/Abstract] OR ADHD[Title/Abstract] OR autism[Title/Abstract] OR trauma[Title/Abstract] OR personality[Title/Abstract] OR thyroid[Title/Abstract] OR sleep[Title/Abstract] OR substance[Title/Abstract]) AND ("1900/01/01"[Date - Publication] : "2026/07/31"[Date - Publication])
```

Mechanisms and biomarkers

Records returned: **1,168**

```
("treatment-resistant depression"[Title/Abstract] OR "treatment refractory depression"[Title/Abstract] OR "refractory depression"[Title/Abstract] OR "Depressive Disorder, Treatment-Resistant"[Mesh]) AND (glutamate[Title/Abstract] OR inflammation[Title/Abstract] OR neuroplasticity[Title/Abstract] OR "HPA axis"[Title/Abstract] OR circadian[Title/Abstract] OR metabolic[Title/Abstract] OR biomarker*[Title/Abstract] OR pharmacogenom*[Title/Abstract]) AND ("1900/01/01"[Date - Publication] : "2026/07/31"[Date - Publication])
```

Treatment

Records returned: **4,273**

```
("treatment-resistant depression"[Title/Abstract] OR "treatment refractory depression"[Title/Abstract] OR "refractory depression"[Title/Abstract] OR "Depressive Disorder, Treatment-Resistant"[Mesh]) AND (augmentation[Title/Abstract] OR switch*[Title/Abstract] OR lithium[Title/Abstract] OR antipsychotic*[Title/Abstract] OR ketamine[Title/Abstract] OR esketamine[Title/Abstract] OR ECT[Title/Abstract] OR rTMS[Title/Abstract] OR "vagus nerve stimulation"[Title/Abstract] OR "deep brain stimulation"[Title/Abstract]) AND ("1900/01/01"[Date - Publication] : "2026/07/31"[Date - Publication])
```

Suicide and mortality

Records returned: **923**

> ("treatment-resistant depression"[Title/Abstract] OR "treatment refractory depression"[Title/Abstract] OR "refractory depression"[Title/Abstract] OR "Depressive Disorder, Treatment-Resistant"[Mesh]) AND (suicid*[Title/Abstract] OR "self-harm"[Title/Abstract] OR mortality[Title/Abstract]) AND ("1900/01/01"[Date - Publication] : "2026/07/31"[Date - Publication])

Psychological and functional factors

Records returned: **1,160**

> ("treatment-resistant depression"[Title/Abstract] OR "treatment refractory depression"[Title/Abstract] OR "refractory depression"[Title/Abstract] OR "Depressive Disorder, Treatment-Resistant"[Mesh]) AND (psychotherap*[Title/Abstract] OR "cognitive behavio*"[Title/Abstract] OR rumination[Title/Abstract] OR anhedonia[Title/Abstract] OR avoidance[Title/Abstract] OR functioning[Title/Abstract] OR "quality of life"[Title/Abstract]) AND ("1900/01/01"[Date - Publication] : "2026/07/31"[Date - Publication])

Selection and Synthesis

Eligible sources were English-language human studies, systematic reviews, meta-analyses, evidence-based guidelines, randomized trials, and large observational or registry studies that directly informed a prespecified domain. Exclusions comprised animal-only studies, case reports, opinion pieces without relevant evidence, duplicate or substantially overlapping reports without unique data, and publications without a clear connection to TRD or a designated differential/maintaining factor. Major-depression evidence not rigorously defined as TRD was retained only when it informed a mechanism or clinical question and was interpreted as indirect evidence.

Davoud Amiri and **Swetang J Shah** independently assessed titles/abstracts and, when required, full texts. Disagreements were resolved through discussion and consensus. Reference lists of relevant reviews, guidelines, and pivotal trials were also examined. Ninety-six distinct sources were included in the final narrative synthesis.

Section-Level Source Counts

Manuscript domain	Distinct cited sources
Diagnostic reassessment	55
Neurobiology	28
Treatment	54
Suicide risk	15
Biomarkers and precision psychiatry	17

Note: *The six PubMed strategies intentionally overlap, so their record counts must not be summed as a unique-record total. Sources may also contribute to more than one manuscript domain; therefore, section-level counts exceed the 96 distinct sources in the final reference list. No PRISMA-style unique-record flow or de novo meta-analysis was conducted because this was a structured narrative review.*
